# Building Intelligent Communication Systems for Handicapped Aphasiacs

**DOI:** 10.3390/s100100374

**Published:** 2010-01-05

**Authors:** Yu-Fen Fu, Cheng-Seen Ho

**Affiliations:** 1 Department of Computer Science and Information Engineering, National Taiwan University of Science and Technology, No. 43, Section 4, KeeLung Road, Taipei 106, Taiwan; 2 Department of Management of Information Systems, China University of Technology, No. 56, Section 3, Shinglung Road, Wenshan District, Taipei 116, Taiwan; 3 Department of Information Technology and Communication, Tuangnan University, No. 152, Section 3, PeiShen Road, ShenKeng, Taipei 22202, Taiwan; E-Mail: shawncs.ho@gmail.com

**Keywords:** handicapped aphasiacs, data glove, finger gestures, finger language, neural network

## Abstract

This paper presents an intelligent system allowing handicapped aphasiacs to perform basic communication tasks. It has the following three key features: (1) A 6-sensor data glove measures the finger gestures of a patient in terms of the bending degrees of his fingers. (2) A finger language recognition subsystem recognizes language components from the finger gestures. It employs multiple regression analysis to automatically extract proper finger features so that the recognition model can be fast and correctly constructed by a radial basis function neural network. (3) A coordinate-indexed virtual keyboard allows the users to directly access the letters on the keyboard at a practical speed. The system serves as a viable tool for natural and affordable communication for handicapped aphasiacs through continuous finger language input.

## 1. Introduction

Disabled people suffering from severe impairments usually face an acute problem: most common interaction modalities are unavailable and their communication capabilities are limited. In fact, it can be very frustrating for them to interact with the computer. They require and expect AAC (Augmentative and Alternative Communication) systems to partly alleviate physical limitations. In general, AAC systems are usually equipped with special access mechanisms, since the users lack the fine motor-control required to operate such systems with standard peripheral devices.

Though an array of modern tools and technologies for AAC has been developed worldwide to assist the disabled, they still have some drawbacks, to name a few:

- Most of them are imported and expensive.
- They are not tailored to local socio-cultural contexts.
- The target users lack necessary motor control to operate associated peripherals.

The objective of this work is to develop an intelligent and easy-to-use communication system (including proper hardware and software) that makes it possible for handicapped aphasiacs to perform basic communication tasks naturally and affordably.

### 1.1. Finger Language

Sign language is popularly used in the deaf-mute circle, but the vocabularies are only a small set of Mandarin and the deaf-mute find it hard to express themselves accurately in sign language. Sign language is utterly difficult for handicapped aphasiacs. It involves too many large-scale movements including combined expressions of fingers, palms, and even arms. For severely handicapped sufferers, this language system is simply too far off in terms of their needs.

Compared with the well-developed sign language scheme, the finger language approach is still at a burgeoning research stage. It originally came from a concept developed at the Southwest Research Institute in 1993 [1] in which a mechanical hand was proposed for the general deaf-and-blind. The mechanical hand was claimed to be able to convert the English alphabet to proper hand gestures. Based on the concept, the Stanford Mechanical Department team led by Dr. Gilden pioneered the 1st communicating mechanical hand, DEXTER, which later had evolved from DEXTER-I to DEXTER-IV models. The DEXTER series made it possible to convert the 26 letters of the English alphabet to hand gestures from keyboards, computers, TV, and telecommunication devices. By their systems, even deaf-and-blind people then became capable of ‘reading’ by touching the gesture-varying mechanical hand [2].

The idea of gestures for pronunciation was first proposed by Lyon [3]. It allows the user to type words or sentences by making the gestures corresponding to the contained syllables. On this basis, the Hanyu Pinyin System was proposed as the Chinese phonetic symbol set in China. Researchers in China use CAS-gloves, cyber-gloves, or colored gloves to conduct language translation from 30 basic hand gestures, designed for pronunciation and named finger-spelling (or manual alphabet or dactylology), into the simplified form of Chinese characters, including the English alphabet and four special consonants: ZH, CH, SH, and NG [4,5]. However, their studies still use the positions and movement orbits in three-dimensional space of both hands. Due to the great differences in culture, local language habits, and even pronunciation, their developed systems are not entirely applicable in Taiwan, which uses the traditional more complicated form of Chinese characters instead of the simplified form as used in mainland China. In addition, our purpose strongly emphasizes the very limited functionality of the handicapped aphasiacs. We need a system that can detect very weak signals caused by very small finger movements and successfully decode the meaning. This requirement is very different from the traditional finger spelling.

### 1.2. Virtual Keyboard

Efficient text entry in AAC systems is very important in helping the disabled to produce statements as easily and quickly as possible during conversation, since they have great difficulty in back-and-forth motions. Many approaches have been proposed. For example, the Chord Keyboard can help the disabled as long as they hit a large button or stir a chord [6], even when he/she is in motion or on bumpy roads. Demasco and McCoy worked on the line-of-gaze technique that tracks eye motion by video camera [7]. The patient makes a direct selection by staring somewhere on a large digitizing tablet. Manaris, *et al.* considered a listening keyboard as a speech input interface for patients [8]. There is a Japanese text input method for a small 12-button mobile phone keypad [9].

Most virtual keyboards are based on the physical layout of a standard keyboard. Before the user can select anything on the screen, he needs some physical instruments for a direct interaction. One of the most essential devices for accessing a computer is the keyboard. People who have no fine motor control can resort to the scanning mechanism associated with the keyboard. The scanning technology moves the cursor row by row until the user selects the desired one by a key or a switch. However, it is difficult for the handicapped aphasiacs to do this in a timely way.

### 1.3. Data Gloves

Considering that the most natural way to operate a computer is by body gestures, some special hardware device that can help the system to extract the meaning of the body gestures is necessary. The fact that the finger language designed for disabled aphasiacs involves only very tiny finger movements imposes very stringent requirements on both the software and hardware of any prospective systems. In this system, we advocate the use as the input device of a data glove that is capable of sensing the slightest movement. Although there are already several imported data gloves of the optic fiber sensor type, they are overly complicated and costly for the intended finger language applications. For example, a data glove from various companies like VPL Research Inc., or 5 DT Inc. may cost from US$2,700 to US$895. This fact has motivated the authors’ initial attempt to make a reliable but low cost (under US$150) data glove.

As past experience points out, the major problems with most finger systems are two fold. One is system reliability and accuracy; the other is the differences in the movements of each individual patient. To overcome such problems, besides the data glove, establishing a delicate tiny-movement-discerning software system is indispensable.

## 2. The Approach

To avoid eavesdropping, ancient Chinese businessmen often settled a transaction by performing specific gestures inside spacious sleeves. They called it “the universe inside the sleeves”, which could be dubbed the earliest figure finger language. We borrow from this idea and use figure gestures as the basic input symbols to develop a communication system for handicapped aphasiacs. Our intelligent communication system involves three key concepts: a data glove to input finger language components, a finger language recognition subsystem to recognize the finger language components, and a virtual keyboard to produce text from the finger language components. The center concept is finger language components. We have defined 12 finger language components in terms of proper finger gestures to support the system.

### 2.1. Data Glove as the Input Device

**Figure 1.**
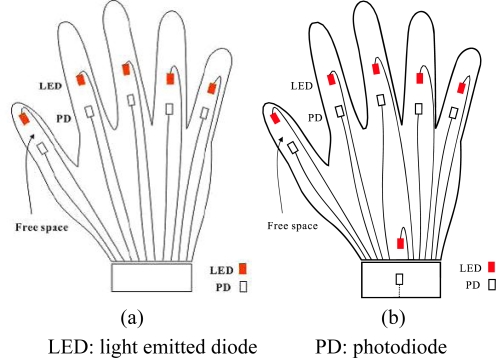
(a) Five sensor data glove design. (b) Six sensor data glove design.

We have manufactured a data glove that uses only light-emitting diodes (LED) and photo-detectors (PD) (*i.e.*, without optic fibers, light from LEDs will reach PDs on line of sight) in measuring the bending degrees of fingers, as illustrated in Figure 1a [10]. The developed data glove is coupled with a virtual keyboard simulated as a predetermined 15 × 6 matrix keying system, which can display the intended message on a screen. Specifically, the x and y coordinates on the virtual keyboard are decided by the movements of the index and middle finger, respectively. Once the desired letter is highlighted, the user then bends his thumb to give the confirmation signal to complete a letter selection sequence. The system described above has two drawbacks and this work proposes two corresponding solutions, as explained below.

- The user needs to carefully move the fingers along the axes to select the desired letter and to make prompt affirmation response (by bending the thumb) when the cursor moves to the letter he intends. This requirement usually involves rather delicate finger movements. It is unrealistic that we assume a handicapped aphasiac is capable of this ability. Our solution is to completely discard the finger-movement-controlled cursor design. Instead, we allow the user to designate a number by his gesture and then use the number to directly move the cursor to the correct position of a letter. In such a way, the user can choose a most becoming speed to enter the text without stress.
- It cannot provide a suitable environment for the user to do continuous finger language input as discussed in [11]. For continuous input, we need to guarantee that between the inputs of two consecutive gestures, we always can successfully distinguish them without mistaking the transition to some other gestures. That is, while the fingers are transforming to a new gesture type, we cannot produce error judgments at any rate. Our solution is to add an extra sensor in the wrist area as shown in Figure 1b, which works as a delimiter corresponding to the bending of the wrist to confirm a gesture input. Our experience suggests that we could correctly set a standard default value to determine whether a hand wrist is bent without learning.

### 2.2. Finger Language Recognition Subsystem

**Figure 2.**
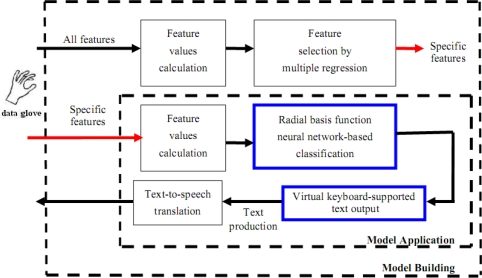
Techniques used in the system.

Given the fact that the finger situations for handicapped aphasiacs are far more complicated and varying than those of normal people, the traditional dichotomy model distinguishing merely the bending and stretching states is inadequate. To fulfill practical needs, gray box modeling is adopted instead, in which the concept of fuzzy sets is incorporated to address the issue of meaning impreciseness, as is true of facing the ubiquitous vague or imprecise statements in real-world linguistic information. Important techniques involved in the recognition of finger language components are shown in Figure 2, including feature values calculation, feature selection and radial basis function neural network-based classification. The major purpose is that given a finger gesture, this subsystem should determine the features of the gesture and then use them to correctly classify it into one of the 11 classes, which correspond to the 11 language components as we defined in Table 1.

#### A. Feature Values Calculation

**Figure 3.**
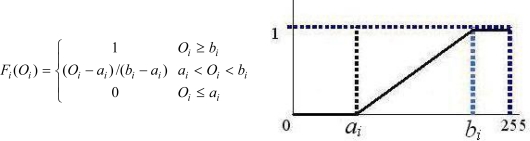
Bending degree function *F_i_*.

This calculates the corresponding values for the input features. Possible features that may play important roles in recognition of finger language are analyzed below along with the method to calculate their values:

- *O_i_*: Original optical signal strength of each finger
  *O_i_* takes on values on the scale of 0 to 255.
- *F_i_*: Bending degree of each finger
  Equation 1 below defines *F_i_* to be a function of *O_i_* to calculate the bending degree for finger *i*.
  (1) $$F_{i}(O_{i})=\{\begin{array}{cc} 1 & O_{i}\geq b_{i}\\ \left(O_{i}-a_{i})/(b_{i}-a_{i}\right) & a_{i}<O_{i}<b_{i}\\ 0 & O_{i}\leq a_{i} \end{array}$$
  where *a_i_* and *b_i_* denote the signal values for the least bent and the least straight situations of finger *i*, respectively. The function is illustrated in Figure 3. Table 2 lists the respective a_i_ and b_i_ values for the five fingers of User4 (Row 4, Table 3). Take *F*_1_ (bending degree function for thumb) as an example. Then, at some specific *O*_1_, we have a corresponding bending degree *F*_1_, as illustrated by Equation 2.
  (2) $$F_{1}(O_{1})=\{\begin{array}{cc} 1 & O_{1}\geq 240\\ \left(O_{1}-80)/(240-80\right) & 80<O_{1}<240\\ 0 & O_{1}\leq 80 \end{array}$$
- *Ni*: Normalized value of original optical signal strength of each finger
  Equation 3 below defines Ni in terms of *O_i_*.
  (3) $$N_{i}=O_{i}/255$$

#### B. Feature Selection

We use a regression technique to select the features. Suppose an output finger language component is represented by a value *Y*. Taking all the features described above, *i.e.*, *O_i_*, *F_i_*, and *N_i_*, into account, then *Y* can be mathematically expressed as Equation 4:

(4) $$\begin{array}{l} Y=C_{0}+(C_{1}\times O_{1}+C_{2}\times O_{2}+C_{3}\times O_{3}+C_{4}\times O_{4}+C_{5}\times O_{5})+(C_{6}\times F_{1}+C_{7}\times F_{2}+C_{8}\times F_{3}+C_{9}\times F_{4}+C_{10}\times F_{5})\\ \;\;+(C_{11}\times N_{1}+C_{12}\times N_{2}+C_{13}\times N_{3}+C_{14}\times N_{4}+C_{15}\times N_{5}) \end{array}$$

where *C_i_*’s with *i* = 0,1,2,…,15, are coefficients to be determined.

We have applied the backward stepwise procedure of multiple regression analysis to the data produced by User 4 (Row 4, Table 3). The procedure begins with all the variables assumed to be relevant to the response. It proceeds by eliminating one least significant variable at a time (at each step) and continues the process until no variables can be removed according to some elimination criterion based upon discriminant function analysis [12]. Eventually, it is found that *O_i_* and *N_i_* are largely statistically insignificant and can be neglected. In Table 3, we also show the customized finger features for ten users. In general, the bending degree features (*F_i_*) are most significant; they are required for almost every user. Some patients did need other features to obtain a better result, among which *N*_2_ and *N*_5_ are most significant.

With this, the above formula for a specific user can be secured as Equation 5:

(5) $$Y=C_{0}+C_{6}\times F_{1}+C_{7}\times F_{2}+C_{8}\times F_{3}+C_{9}\times F_{4}+C_{10}\times F_{5}$$

The goodness of fit (R2) of Equation 5 is calculated to be 0.99, 0 ≤ R2 ≤ 1, which means using this regressed equation can successfully explain 99% of variance of *Y*, that is to say, Equation 5 has high goodness of fit.

#### C. Radial Basis Function Neural Network-Based Classification

**Figure 4.**
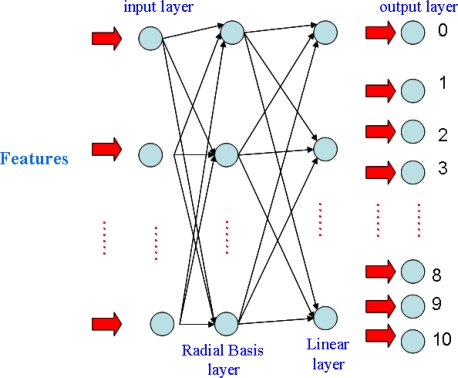
Radial basis function neural network-based classification.

We are employing the radial basis function neural network (RBFNN) for classification. The architecture, illustrated in Figure 4, learns how to classify finger language components according to the selected input features. The selected feature values calculated in the above are first input at the input layer of the network, and after going through manipulations in the hidden layer neurons, 11 state values come out of the output layer, which correctly identify the corresponding components. Our implementation tool is MATLAB.

Initially the hidden layer has no neurons; the neurons are then added one by one until the network's mean squared error falls below 0.001. In our case, the number of neurons in hidden layer is settled to 93 for the user under experiment.

### 2.3. Virtual Keyboard-Supported Text Output

Shown also in Figure 2 is the virtual keyboard that supports the text output. Our virtual keyboard is so designed that a handicapped aphasiac can create text from the keyboard on the screen by wearing the data glove to directly select the letters on the keyboard. The virtual keyboard is a two dimensional array of size 7 × 7. We use the 12 language components to manipulate the keyboard, including Component 0 till Component 10 as illustrated in Table 1 and an extra Component 11 representing wrist bending. Specifically, Component 1 up to Component 7 is used to designate the coordinates X and Y of a cell on the keyboard to access the letter in the cell. Component 8 is used to erase an incorrect gesture input and signal a new gesture input to follow. Component 11 works as a delimiter to confirm a coordinate input so that another coordinate input can correctly follow. Finally, Components 0 and 9 are used to designate input language, *i.e.*, English or Chinese, respectively. The layouts for English and Chinese keyboards are respectively shown in Figures 6 and 7. For example, to input English letter E (cell (5, 1) on the English keyboard), a sequence of four components are used: Component 5 (X coordinate of E), Component 11, Component 1 (Y coordinate of E), and finally Component 11, as illustrated in Table 4. By this way, a handicapped aphasiac can correctly enter any sequences of letters to compose his text. Moreover, the dwell time of the key input can be set individually to reflect personal taste. In a word, the virtual keyboard can successfully extend our previous static finger language recognition capability [13] into a continuous finger language recognition system and creates an intelligent text input environment for handicapped aphasiacs.

Note that the English virtual keyboard looks rather different from the traditional layout. The letters are arranged according to the alphabetical sequence as shown in Figure 6. This is because our input method has nothing to do with the statistics about how often letters are used in composing words and it

**Figure 5.**
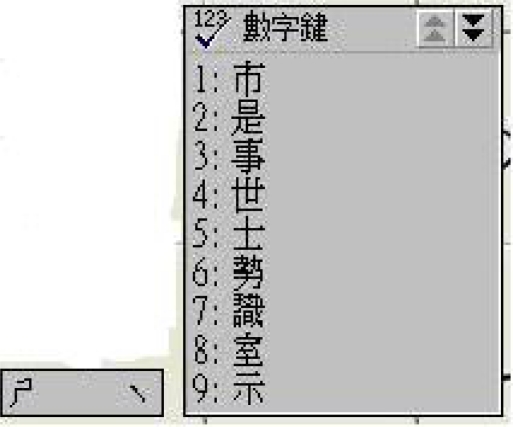
The homophones of an input phonetic symbol.

**Figure 6.**
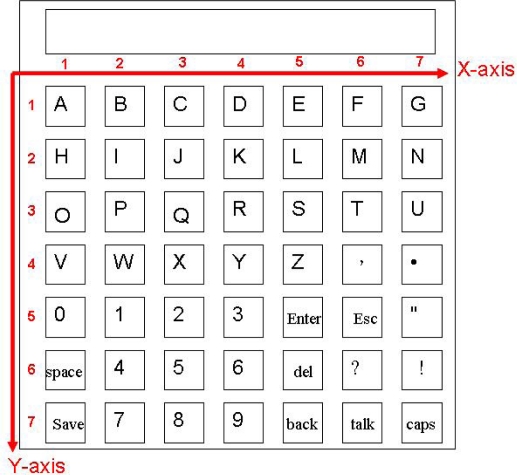
English virtual keyboard.

**Figure 7.**
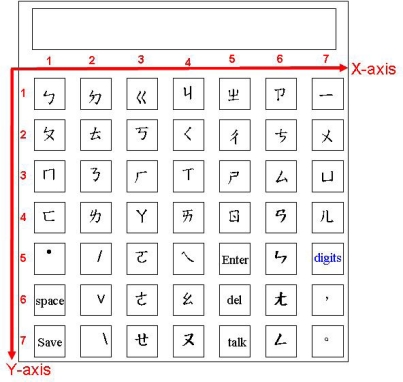
Chinese virtual keyboard.

It costs the same to input any letter by designating the coordinates X and Y of the corresponding cell. The Chinese virtual keyboard is specifically developed for Taiwanese users for they use the Taiwan phonetic system to enter words. The keyboard supports the 37 phonetic symbols, the 5 tone symbols—short tone (•), tone one (space), tone two (/), tone three (v), and tone four (\), the conjugate characters, and the numeric characters. Characters like space, backspace, and carriage return are also supported. When a user inputs a phonetic symbol, e.g., 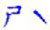, the system will list all the homophones on an associated menu, as illustrated in Figure 5. The user then uses components 1 to 9 to choose the correct word. Component 10 works for “Page down” by turning to the next page when the homophones span more than one page. Component 11 is used to ‘Confirm the choice’ as before. The number keys on the Chinese keyboard are for continuous digit input, for example, telephone number.

This virtual keyboard design helps a patient enter text without fooling around his hands over the keyboard. Instead, he can select a most suitable speed for text input without being forced to make delicate movements and prompt responses from AAC facilities.

## 3. System Development and Application

A typical scenario of system development for a specific user is illustrated as follows:

Step 1: Adjustment of the glove: The user adjusts PDs and LEDs associated with the five fingers, respectively, so that for each finger a dynamic range of the grayscale covering 0 to 255 (or, maximally achievable) can be secured.

Step 2: Tailoring of values a and b: The values of a and b are specifically tailored for the user by Equation 6, where *i* = 1, 2, 3, 4, 5 stands for finger *i* of the user.

(6) $$\{\begin{array}{c} a_{i}=\text{max}(\text{optical}\;\text{intensity}\;\text{when}\;\text{finger}\;i\;\text{is}\;\text{bent})\\ b_{i}=\text{min}(\text{optical}\;\text{intensity}\;\text{when}\;\text{finger}\;i\;\text{is}\;\text{straight}) \end{array}$$

Step 3: Creation of training data: The user is instructed to make static finger items designating numbers 0, 1, 2, to 10 and 100 times each, in an attempt to cover the widest variations in finger signal detection. In the end, each user should produce 11 × 100 = 1,100 records of data.

Step 4: Feature selection: The features specifically working best for the users are decided by multiple regression analysis applied on the above training data.

Step 5: Model training: The training data in Step 3 that are related to the selected features are singled out as the training data set and fed into the neural network, which eventually learns a radial basis function for each hidden neuron. After the stratified tenfold cross validation, the neural network finalizes to the best recognition model and is ready for use.

Table 5 shows the results of stratified tenfold cross validation for the ten users, showing 100% of correctness. At this step, we can make sure the customized static finger language recognition function works well. Note that the customization is required because we find each handicapped aphasiac has his unique finger positions, sizes, shapes, and cross-finger-influence while bending fingers. Now we can turn the system into a continuous finger language recognition system with the support of the two dimensional on-screen keyboard.

Now the system can be put in everyday use by the user:

Step 1: Virtual keyboard adjustment: At this step, the subject is trained to get familiar with the manipulation of the data glove plus the on-screen virtual keyboard so that he feels comfortable with the controlling of a computer from his AAC system.

Step 2: Text production: The user puts on the glove and makes gestures; the recognition system recognizes the corresponding language components and the virtual keyboard displays the designated letters. The letters finally develop into a sentence.

Step 3: Speech production: The intended sentence is finally sent to the text-to-speech module for speech production.

## 4. Summary and Discussions

This paper describes an intelligent communication system that can work as a general mode of communication for handicapped aphasiacs in Taiwan. It produces text for communication by employing three key techniques: a data glove to input finger language components, a finger language recognition subsystem to recognize language components, and a virtual keyboard to produce text from language components. A handicapped aphasiac can enter text via the virtual keyboard by wearing a data glove to make proper finger gestures and use them to directly select the letters on the keyboard.

Some interesting features of the system are as follows. First, the system is customizable by allowing easy adjustment of the data glove to make it fit to specific handicapped aphasiacs. It employs a function, the shape of which is easily adjustable for each finger, to effectively calculate the bending degrees of the fingers. It employs multiple regression analysis to automatically extract proper finger features so that the recognition model can be fast and correctly constructed by a radial basis function neural network. Finally, the coordinate-indexed virtual keyboard works as a continuous text input media, allowing the users to access the letters on the keyboard with a most becoming speed.

The major contribution of the work is that the virtual keyboard can be considered as a valuable low-cost enhancement to the data glove to effectively help handicapped aphasiacs. We have demonstrated that efficient and effective manipulation of the virtual keyboard using finger-language-based interaction techniques is possible. This will greatly enhance the possibilities for physically-challenged users to work, on a more equitable basis, with their able-bodied colleagues with the help of the same software products.

This work has opened a possibility for us to extend our research to the recognition of dynamic finger gestures in the future, which allows more real applications of the system. As a matter of fact, the virtual keyboard, arranged by the alphabetical order, has been tested and confirmed by several users to be suitable for typing letters. Another further extension is to improve the customization process by pre-developing a “pseudo-general” finger language recognition system with the support of the development of a database that accommodates the finger gestures from comprehensive representative handicapped aphasiacs. With this, we then can quickly create a customized system for a specific user by performing a slight adjustment to the system.

## Figures and Tables

**Table 1.**
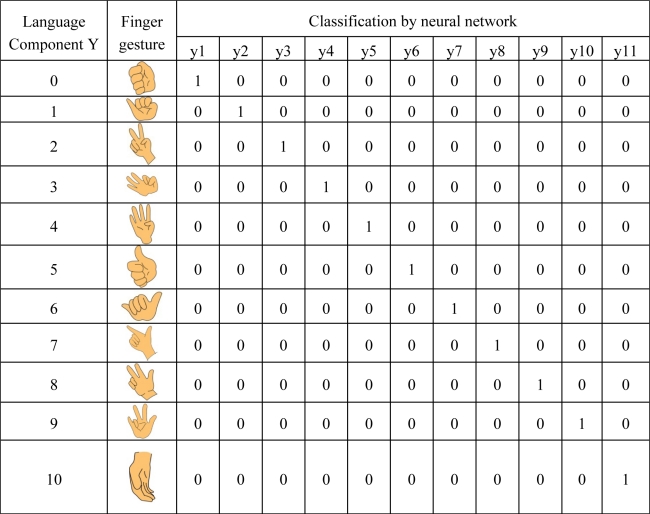
Finger language components needing learning.

**Table 2.** Values *a* and *b*-parameterized bending degree function.

*F*_1_: Thumb		*F*_2_: Index-finger		*F*_3_: Middle-finger		*F*_4_: Ring-finger		*F*_5_: Little-finger	
*a_1_*	*b_1_*	*a_2_*	*b_2_*	*a_3_*	*b_3_*	*a_4_*	*b_4_*	*a_5_*	*b_5_*
80	240	90	240	80	190	70	180	60	240

**Table 3.** Feature selection.

	**Features**	**Goodness of fit (R2)**

User1	F_1_ – F_5_, N_1_, N_2_	0.99
User2	F_1_ – F_5_, N_2_, N_3_	0.99
User3	F_1_ – F_5_, N_1_, N_2_, N_4_, N_5_	0.99
User4	F_1_ – F_5_	0.99
User5	F_1_ – F_5_, N_1_ – N_5_	0.99
User6	F_1_ – F_5_	0.99
User7	F_1_ – F_5_	0.99
User8	F_1_ – F_5_	0.99
User9	F_1_ – F_4_, N_4_, N_5_	0.99
User10	F_1_ – F_5_, N_2_, N_4_, N_5_	0.99

**Table 4.** Exemplified input sequence for letter E (containing 3 steps/5 gestures).

Step	Meaning	Gesture Annotation	Finger Gesture
1	English Input	Gesture: 0	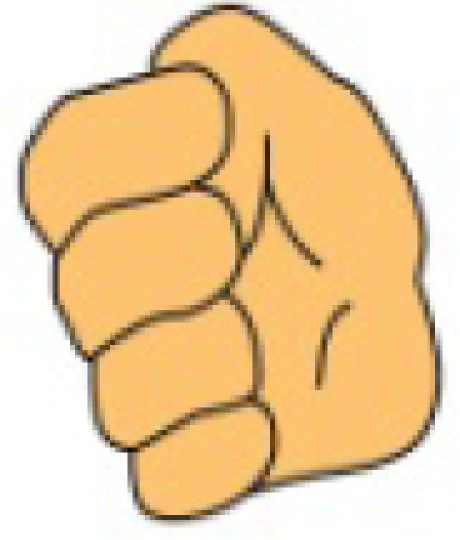
2	X coordinate: 5	First Gesture: 5 Delimiter Gesture	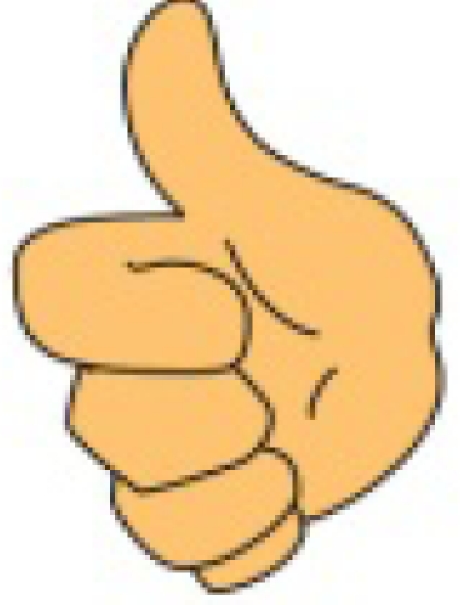 Wrist Bent
3	Y coordinate: 1	Second Gesture: 1 Delimiter Gesture	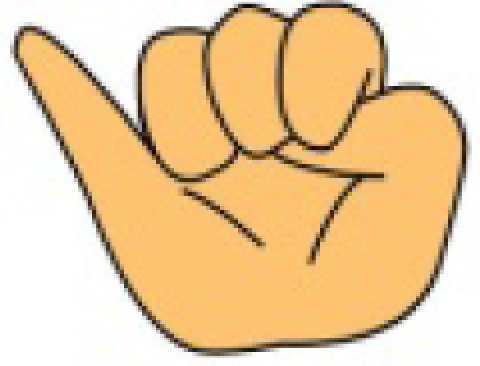 Wrist Bent

**Table 5.** Results of stratified tenfold cross validation.

	**Neural network**	**Features**	**Error in each gesture**										

			**0**	**1**	**2**	**3**	**4**	**5**	**6**	**7**	**8**	**9**	**10**

User1	RBFNN	F_1_ – F_5_, N_1_, N_2_	0	0	0	0	0	0	0	0	0	0	0
User2	RBFNN	F_1_ – F_5_, N_2_, N_3_	0	0	0	0	0	0	0	0	0	0	0
User3	RBFNN	F_1_ – F_5_, N_1_, N_2_, N_4_, N_5_	0	0	0	0	0	0	0	0	0	0	0
User4	RBFNN	F_1_ – F_5_	0	0	0	0	0	0	0	0	0	0	0
User5	RBFNN	F_1_ – F_5_, N_1_– N_5_	0	0	0	0	0	0	0	0	0	0	0
User6	RBFNN	F_1_ – F_5_	0	0	0	0	0	0	0	0	0	0	0
User7	RBFNN	F_1_ – F_5_	0	0	0	0	0	0	0	0	0	0	0
User8	RBFNN	F_1_ – F_5_	0	0	0	0	0	0	0	0	0	0	0
User9	RBFNN	F_1_ – F_4_, N_4_, N_5_	0	0	0	0	0	0	0	0	0	0	0
User10	RBFNN	F_1_ – F_5_, N_2_, N_4_, N_5_	0	0	0	0	0	0	0	0	0	0	0
